# Epidemiology and Clinical Relevance of *Pneumocystis jirovecii* in Non-Human Immunodeficiency Virus Patients at a Tertiary Care Center in Central Europe: A 3-Year Retrospective Study

**DOI:** 10.3390/jcm14082820

**Published:** 2025-04-19

**Authors:** Ágnes Jakab, Andrea Harmath, Zoltán Tóth, László Majoros, József Kónya, Renátó Kovács

**Affiliations:** 1 Department of Medical Microbiology, Faculty of Medicine, University of Debrecen, Nagyerdei krt. 98., 4032 Debrecen, Hungary; jakab.agnes@med.unideb.hu (Á.J.); harmath.andrea@med.unideb.hu (A.H.); toth.zoltan@med.unideb.hu (Z.T.); major@med.unideb.hu (L.M.); konya@med.unideb.hu (J.K.); 2 Medical Microbiology, Clinical Centre, University of Debrecen, Nagyerdei krt. 98., 4032 Debrecen, Hungary; 3 Doctoral School of Pharmaceutical Sciences, University of Debrecen, Nagyerdei krt. 98., 4032 Debrecen, Hungary

**Keywords:** *Pneumocystis jirovecii*, (1-3)-β-D-glucan, HIV, epidemiology

## Abstract

**Background/Objectives:** This study examines the clinical characteristics of *Pneumocystis jirovecii* pneumonia (PjP) in non-Human immunodeficiency virus (HIV) patients in Hungary to describe its local epidemiological properties. **Methods**: Our study was conducted at a clinical center with more than 1700 beds at the University of Debrecen in Hungary. We included all patients without HIV infection for whom a diagnostic evaluation for *Pneumocystis* infection had been requested between 1 January 2022 and 31 December 2024. **Results**: In total, 21 cases of PjP were identified from 122 requests at the University of Debrecen Clinical Center between 2022 and 2024. The overall 30-day mortality rate was 43% in PjP. Admission to the intensive care unit (odds ratio [OR] 5.44, 95% confidence interval [CI] 1.87–14.09, *p* = 0.001), the need for mechanical ventilation (OR 4.09, 95% CI 1.45–12.14, *p* = 0.015) and hematological malignancies (OR 3.24, 95% CI 1.23–9.18, *p* = 0.024), were associated with *Pneumocystis* PCR positivity. Furthermore, a significant association was observed between elevated levels of C-reactive protein (OR 1.01, 95% CI 1–1.01, *p* = 0.001), 30-day mortality (OR 2.86, 95% CI 1.09–7.92, *p* = 0.049), and *Pneumocystis* PCR positivity. Regarding diagnostic platforms used, Fujifilm Wako assay detected serum (1-3)-β-D-glucan positivity (>7 pg/mL) from 352 copies/mL in non-HIV patients with probable PJP. **Conclusions**: Our study serves as a gap-filling investigation, providing an overview of *Pneumocystis* epidemiology in the Central European region.

## 1. Introduction

*Pneumocystis jirovecii* is an opportunistic fungal pathogen, classified as a medium-priority pathogen in the fungal priority list published by the World Health Organization [1]. Predisposing factors for *P. jirovecii*-related pneumonia (PjP) include transplantation, hematological malignancies, inflammatory or rheumatologic conditions, and related therapies that impair cell-mediated immunity [2,3,4]. PjP is no longer restricted to Human immunodeficiency virus (HIV)-positive patients but is increasingly diagnosed in non-HIV populations, posing new challenges for diagnosis and treatment [2,3,4]. In case of HIV-positive patients, the onset of PJP is usually gradual and insidious with limited radiologic findings, while in immunocompromised non-HIV individuals, clinical presentation tends to be more acute with rapid emergence of respiratory symptoms and with a mortality rate twice that of HIV-infected individuals, ranging from 30% to 60% [4,5,6].

*P. jirovecii* is globally distributed; however, data on its prevalence and incidence in Central and/or Eastern European populations are limited. The incidence of PjP in Central and Eastern European countries has been reported to range from 0.18 to 0.88 per 100,000 admissions [7,8,9]. However, these data usually pertain to HIV-infected patients, and there are no reliable data on the non-HIV population in this region.

Hence, the primary aim of this study was to retrospectively investigate the epidemiological data and the clinical characteristics of *P. jirovecii* infection among HIV-negative patients in one of the largest tertiary care centers in Hungary, thereby enhancing our understanding of *P. jirovecii* infections.

## 2. Materials and Methods

Our study was conducted at a clinical center with more than 1700 beds at the University of Debrecen in Hungary. We included all patients without HIV infection for whom a diagnostic evaluation for *Pneumocystis* infection had been requested between 1 January 2022 and 31 December 2024. In case of PJP diagnosis, we followed the EORTC/MSGERC revised definitions for *P. jirovecii* disease, where the triad of host factors, clinical characteristics, and mycologic tests was considered [10]. The diagnosis of PjP was based on the administration of therapeutic doses of corticosteroid therapy and CD4^+^ lymphocyte count (where it was available); the presence of suggestive clinical criteria including fever, respiratory symptoms (e.g., cough, dyspnea, hypoxemia), bilateral or diffuse ground-glass opacity on X-ray with interstitial infiltrates; and a positive microbiological diagnostic test, including the detection of (1-3)-β-D-glucan in blood and/or a positive polymerase chain reaction (PCR) result from a bronchoalveolar lavage specimen or induced sputum [10] Notably, our laboratory does not perform microscopy-based examinations; therefore, according to the EORTC/MSGERC guideline, we can establish only probable PJP results [10]. In clinical practice, we adhere to the diagnostic algorithm for PJP as outlined in Table 1.

Demographic data, underlying medical conditions, hematological parameters, blood gas parameters, and details of antimicrobial therapy were collected from the patients’ medical records. Concurrent bacteremia and/or fungaemia were defined as the isolation of potentially pathogenic microbes from blood culture samples at the time of *Pneumocystis* infection. PjP outcomes were monitored from the initial diagnosis until 30 days post-diagnosis or death. Regarding *Pneumocystis* laboratory diagnosis, copy numbers and serum (1-3)-β-D-glucan levels were obtained using the *Pneumocystis* ELITe MGB^®^ Kit (Elitech Group SAS, Puteaux, France) and the Fujifilm Wako assay (FUJIFILM Wako Pure Chemical Corporation, Osaka, Japan), respectively. The limit of detection of polymerase chain reaction is <97 copies/mL, while the cut-off value of Fujifilm Wako assay is 7 pg/mL.

Univariable analysis was performed to reveal those factors, which are associated with PCR positivity. Categorical variables were analyzed using Fisher’s exact test. In the case of continuous variables, a logistic regression model was used and based on the distribution of the data, the Mann–Whitney test was used for non-normally distributed variables. Data analysis was performed using GraphPad Prism software (version no.: 10.1.1). Results were considered significant if the *p*-value was <0.05.

## 3. Results

In total, 122 requests for *P. jirovecii* diagnosis were registered from non-HIV patients during the investigation period, of which 21 were probable PJP according to EORTC/MSGRC guidelines [10]. PjP diagnosis was based on bronchoalveolar lavage fluid positivity in 33% of cases (seven lavage samples were PCR-positive). In eight patients, the diagnosis was based on bronchial specimen positivity, while six patients had a positive induced sputum sample. The number of requests increased continuously, with 18, 31, and 73 registered in 2022, 2023, and 2024, respectively. Notably, the increase in the number of requests is not specific to *Pneumocystis*; it reflects a general trend across all areas of microbiology in the post-COVID-19 era at our clinical center. The demographic and clinical characteristics, treatment, and outcomes of *Pneumocystis*-positive patients, as well as the results of laboratory tests performed, are presented in Table 2.

Intensive care unit admission, invasive mechanical ventilation, and hematological malignancy were observed in 52%, 33%, and 43% of patients with positive *Pneumocystis* PCR results, respectively. The majority of patients (79 out of 122 [65%]) were male, and the median age was 61 years (range: 3 to 98 years). Based on the results of univariable analysis, intensive care unit admission (OR 5.44, 95% CI 1.87–14.09, *p* = 0.001), invasive mechanical ventilation (OR 4.09, 95% CI 1.45–12.14, *p* = 0.015), hematological malignancy (OR 3.24, 95% CI 1.23–9.18, *p* = 0.024), and 30-day mortality (OR 2.86, 95% CI 1.09–7.92, *p* = 0.049) were significantly associated with *Pneumocystis* PCR positivity (Table 1).

Focusing on the applied therapies, the use of glucocorticoids, chemotherapeutic agents, and monoclonal antibodies has been shown to significantly increase the risk of PJP [11]. In our study, corticosteroid therapy (OR 15.44, 95% CI 2.49–164.2, *p* = 0.0004), intravenous antibiotic use (OR 7.14, 95% CI 2.05–23.86, *p* = 0.0007), sulfamethoxazole/trimethoprim therapy (OR 21.00, 95% CI 6.12–61.00, *p* < 0.0001), chemotherapeutic agents (OR 4.50, 95% CI 1.59–11.75, *p* = 0.007), and monoclonal antibody treatment (OR 7.68, 95% CI 2.12–26.13, *p* = 0.0034) were significantly associated with positive *Pneumocystis* PCR results (Table 2).

Among PCR-positive cases, 12 patients (57%) presented with fever, although its presence and severity may have been influenced by concurrent therapies. Furthermore, all PCR-positive patients exhibited bilateral or diffuse ground-glass opacities with interstitial infiltrates on chest X-ray. Notably, none of the *Pneumocystis*-positive patients had undergone solid organ or hematopoietic stem cell transplantation. CD4^+^ cell count data were available for 11 PCR-positive patients (52%), of whom four died. The mean CD4^+^ cell count was 1005 ± 491 cells/mm^3^, with a range of 120 to 1740 cells/mm^3^.

Bacterial and/or fungal bloodstream co-infections were reported in 13 (11%) cases and 3 (2%) cases, respectively. It is noteworthy that concomitant bacteraemia (OR 3.63, 95% CI 1.15–11.5, *p* = 0.047) was associated with positive *Pneumocystis* PCR results (Table 2). Bacterial and/or fungal respiratory co-infections were present in 38% of PjP cases, including *Escherichia coli* (two cases), *Staphylococcus aureus* (three cases), *Klebsiella pneumoniae* (three cases), and *Pseudomonas aeruginosa* (one case). Among the laboratory parameters examined, C-reactive protein was significantly elevated in PCR-positive cases compared to PCR-negative cases (*p* = 0.001) (Table 2). Regarding microbiological diagnosis, the median of quantitative PCR copy numbers 195 copies/mL, ranging from 97 to 684,201. Positive serum (1–3)-β-D-glucan levels were detected in 7 of the 21 cases.

## 4. Discussion

Based on large-scale national epidemiological data, there has been a significant increase in the prevalence and incidence of PjP in non-HIV patients [2,3,4,6,12]. This concerning trend is attributed to the extensive use of corticosteroids and the increased implementation of organ and stem cell transplantation [2,3,4,6,12]. In line with previously published studies, the most common immunocompromising conditions observed in our study were hematological and solid malignancies, which is consistent with findings in hospitalized patients with PjP in general [2,13]. In addition, hematological malignancies showed a significant relationship with *Pneumocystis* PCR positivity. In this study, 95% of patients had received corticosteroid therapy—a well-known predisposing factor for PjP [2,3,4]—which, along with chemotherapeutic drugs, was associated with *Pneumocystis* PCR positivity. Notably, the observed 30-day mortality was significantly higher (43%) compared to the HIV-infected population (approximately 10–15%) [5,6,7,8,9,10] and demonstrated a clear correlation with PCR positivity. Focusing on additional risk factors in *Pneumocystis* PCR-positive patients, only 2 of the 11 available CD4^+^ cell count values were below 200. A previously published systematic reviews concluded that a CD4^+^ cell count of less than 200 was a reliable biomarker of “high risk” category in immunocompromised non-HIV patients [14,15]. Nevertheless, higher CD4^+^ cell number does not exclude the possibility of PJP as described by Koifman et al. [16].

In this study, 23% of the patients required admission to the intensive care unit, while 15% received invasive mechanical ventilation. Notably, both factors were significantly more common among patients showing *Pneumocystis* PCR positivity. Schmidt et al. [17] reported that more than 40% of patients required intensive care unit admission, with 36% needing invasive mechanical ventilation. According to previous data, 16% of HIV-positive and 50% to 60% of non-HIV patients require mechanical ventilation during PjP hospitalization [18,19]. Monnet et al. [20] reported a 62% mortality rate among patients who required mechanical ventilation. These previously published findings are consistent with our results.

Although diagnostic tests have improved over the last decade, several laboratory parameters can further support diagnosis. These parameters may differ between HIV-negative and HIV-positive individuals. In our study, C-reactive protein was elevated and was significantly higher among patients who showed a positive *Pneumocystis* PCR result; however, the degree of elevation is generally lower compared to that observed in bacterial infections [21]. Sage et al. [21] demonstrated that HIV-infected patients with PjP showed a significant association between elevated C-reactive protein levels, disease severity, and poor outcomes.

Based on EORTC/MSGERC guideline, the diagnosis of proven PJP is based on clinical and radiological criteria with microscopic visualization of *P. jirovecii* in respiratory specimens [10]. Although, PCR-based platforms are more sensitive than microscopic examination for the detection of *P. jirovecii*, their sensitivity does not support the differentiation between proven PJP and colonization with *P. jirovecii*. In addition, in the HIV-negative immunocompromised population, the differentiation between *P. jirovecii* colonization and active PjP remains further challenging, especially in the intensive care unit where PCR-based diagnostics are commonly used. Previous studies have shown that a significant proportion of PCR-positive cases in these patients may show colonization rather than true infection [22,23]. A multicenter retrospective study involving intensive care unit patients with severe pneumonia described that nearly 40% of those who showed *Pneumocystis* PCR positivity were classified as colonized, not infected. In case of these cases, lower lymphocyte counts and higher rates of viral co-infections (e.g., Cytomegalovirus and Epstein–Barr virus) were observed compared with patients with confirmed PjP [24]. Another major finding of this study was that *P. jirovecii* colonization was an independent predisposing factor for increased 28-day mortality, suggesting the clinical significance of the presence of *P. jirovecii* without active infection [24]. According to EORTC/MSGERC, all nucleic acid amplification tests should be validated in the appropriate clinical context (e.g., non-HIV patients vs. HIV patients) to define the thresholds of colonization and definitive PJP [10]. Quantitative PCR combined with serum (1-3)-β-D-glucan determination may aid in distinguishing disease from colonization. However, because of methodological variability, there is no universally accepted cut-off value to differentiate between the two [3].

In our study, the measured copy numbers could suggest either colonization or infection. Generally, the *P. jirovecii* load is significantly lower in non-HIV patients. Previous studies indicate that positive PCR values below 1450 copies/mL may be associated with both colonization and infection in the HIV-negative population, and patients with low pathogen densities (85 copies/mL) may still have PjP [22,23]. Serum (1-3)-β-D-glucan determination has good sensitivity and a high negative predictive value in HIV-positive patients with PjP. However, its cut-off values are not well defined, and its sensitivity in HIV-positive patients was higher than those without HIV (94% vs. 86%) with similar specificity [25,26]. Jiang et al. (2025) [24] shows that the serum (1-3)-β-D-glucan concentration in patients colonized with *P jirovecii* is lower than in patients with PjP. However, data from several patients with *P jirovecii* colonization were higher than normal values.

In light of these considerations, the differentiation between *Pneumocystis* colonization and active infection remains a significant diagnostic challenge in the present study; nonetheless, the combination of quantitative PCR and serum (1-3)-β-D-glucan determination may result in superior diagnostic performance. In our study, Fujifilm Wako assay detected serum (1-3)-β-D-glucan positivity (>7 pg/mL) from 352 copies/mL in non-HIV patients with probable PJP. As we wrote above, our laboratory did not perform microscopy-based examination during the observation period; therefore, we could not establish a proven diagnosis of *P. jirovecii* infection. Based on our local diagnostic algorithm, real-time PCR is recommended as the principal microbiological diagnostic test for PjP, while serum (1-3)-β-D-glucan testing may be performed as an adjunctive test. A positive real-time PCR result with compatible clinical course and chest X-ray or computer tomography findings is indicative of the definitive diagnosis of PjP. Furthermore, our local algorithm recommends consultation with an infectious disease specialist to differentiate true infection from colonization.

For the sake of completeness, some limitations of this study should be highlighted. First, the analysis was conducted at a single center; therefore, the number of *Pneumocystis*-positive patients was relatively small, limiting the depth of statistical analysis. Second, our laboratory does not perform microscopy-based examination; therefore, according to the EORTC/MSGERC guideline, we can provide only probable PJP results [10]. Furthermore, this guideline wrote that CD4^+^ cell count of less than 200 was a sensitive biomarker of “high risk” in immunocompromised patients without HIV [10]; however, here we could receive this data only from the 52% of involved PCR positive patients, which may undermine the uniformity of case definition. Despite these limitations, this study serves as a gap-filling investigation, providing an overview of *Pneumocystis* epidemiology in the Central European region.

## Figures and Tables

**Table 1:** Diagnostic algorithm used in our laboratory in the absence of microscopy-based investigation.

	Criteria	Interpretation
**Clinical presentation**	Clinical symptoms suggestive of PjP and bilateral or diffuse ground-glass opacity on X-ray with interstitial infiltrates.	Suggest possible PjP infection
**PCR for *P. jirovecii***	Positive PCR result from respiratory sample	Indicates presence of *P. jirovecii* DNA
**Detection of serum (1-3)-β-D-glucan**	Elevated above diagnostic threshold	Suggests fungal infection, supports *P. jirovecii* PCR as adjunctive test
**Final diagnosis**	Clinical signs + Positive PCR (+elevated (1-3)-β-D-glucan)	Probable PJP diagnosis, further expert consultation may be needed

**Table 2:** Microbiological characteristics and clinical variables for *Pneumocystis jirovecii* pneumoniae in HIV-negative patients.

Variables	Total	*Pneumocystis* PCR Positive	*Pneumocystis* PCR Negative	Odds Ratio	95% Confidence Intervals (CI)	*p*-Value
	122 (100%)	21 (17%)	101 (83%)			
**Demographic**						
**Age**						
≤50 years	33 (27%)	6 (29%)	27 (27%)	1.1	0.38–3.05	>0.999
>50 years	89 (73%)	15 (71%)	74 (73%)	0.91	0.33–2.62	>0.999
**Gender**						
Female	43 (35%)	7 (33%)	36 (36%)	0.90	0.35–2.36	>0.999
Male	79 (65%)	14 (67%)	65 (64%)	1.11	0.42–2.84	>0.999
**Clinical presentation**						
**Healthcare-associated risk factors**						
Intensive Care Unit	28 (23%)	11 (52%)	17 (17%)	5.44	1.87–14.09	0.001 ^1^
Days in Intensive Care Unit (median and range)	0 (0–58)	3 (0–33)	0 (0–58)	1.02	0.99–1.06	0.253
Invasive mechanical ventilation	18 (15%)	7 (33%)	11 (11%)	4.09	1.45–12.14	0.015 ^1^
**Underlying comorbidities**						
Autoimmune disease	9 (7%)	2 (10%)	7 (7%)	1.41	0.28–7.48	0.652
Diabetes mellitus	22 (18%)	1 (5%)	21 (21%)	0.19	0.02–1.27	0.118
Renal failure	15 (12%)	2 (10%)	13 (13%)	0.71	0.15–3.34	>0.999
Hematological malignancy	28 (23%)	9 (43%)	19 (19%)	3.24	1.23–9.18	0.024 ^1^
Solid malignancy	28 (23%)	5 (24%)	23 (23%)	1.06	0.39–3.19	>0.999
Chronic obstructive airway disease (COPD)	13 (11%)	3 (14%)	10 (10%)	1.52	0.41–5.27	0.696
**Co-infections**						
Bacteriaemia	13 (11%)	5 (24%)	8 (8%)	3.63	1.15–11.5	0.047 ^1^
Fungaemia	3 (2%)	1 (5%)	2 (2%)	2.48	0.16–21.86	0.436
Adenovirus infection	4 (3%)	1 (5%)	3 (3%)	1.63	0.12–11.38	0.535
Cytomegalovirus infection	4 (3%)	2 (10%)	2 (2%)	5.21	0.76–34.13	0.137
Epstein–Barr virus infection	5 (4%)	1 (5%)	4 (4%)	1.21	0.09–8.06	>0.999
**Treatment**						
Corticosteroid therapy	77 (63%)	20 (95%)	57 (56%)	15.44	2.49–164.2	0.0004 ^1^
Prednisone therapy (≥0.3 mg/kg)	70 (57%)	16 (76%)	54 (53%)	2.79	0.99–7.3	0.088
Receipt of systemic antibiotics	64 (52%)	18 (86%)	46 (46%)	7.14	2.05–23.86	0.0007 ^1^
Sulfamethoxazole/Trimethoprim	34 (28%)	17 (81%)	17 (17%)	21	6.12–61	<0.0001 ^1^
Receipt of systemic antifungal	37 (30%)	8 (38%)	29 (29%)	1.53	0.56–4.01	0.438
Chemotherapeutic drugs	58 (48%)	16 (76%)	42 (42%)	4.5	1.59–11.75	0.007 ^1^
Monoclonal antibodies	11 (9%)	6 (29%)	5 (5%)	7.68	2.12–26.13	0.0034 ^1^
**Mortality**						
30-day mortality	30 (25%)	9 (43%)	21 (21%)	2.86	1.09–7.92	0.049 ^1^
**Laboratory results**						
**Blood parameters (mean with range)**						
White blood cell count (giga/L)	10.2 (0.1–44.4)	10.2 (0.1–32.5)	10.2 (0.6–44.4)	1	0.94–1.07	0.99
Neutrophil granulocyte count (giga/l)	8.1 (0.3–40.6)	7 (0.3–16.8)	8.4 (0.6–40.6)	0.96	0.87–1.05	0.371
Lymphocyte count (giga/L)	1.8 (0.1–29.5)	2.7 (0.1–29.2)	1.5 (0.2–29.5)	1.12	0.94–1.35	0.213
Creatinine (μM/L)	100 (4–766)	115 (27–766)	96 (4–479)	1	1–1.01	0.448
C-reactive protein (mg/L)	89.5 (0.5–507)	156 (1.8–507)	72.9 (0.5–277.2)	1.01	1–1.01	0.001 ^1^
Lactate dehydrogenase (U/L)	296 (2–4863)	369 (37–913)	267 (2–4863)	1	1–1	0.502
**Blood gas parameters (mean with range)**						
Partial pressure of carbon dioxide (pCO_2_) (Hgmm)	41.6 (2.3–66)	36 (2.3–58)	48.2 (26–66)	0.948	0.89–1.01	0.097
Partial pressure of oxygen (pO_2_) (Hgmm)	54.2 (2.3–90)	52.8 (2.3–89)	55.9 (29–90)	0.995	0.96–1.03	0.775
Bicarbonate (HCO_3_) (mmol/L)	27 (16–41.2)	27.1 (16.7–40.3)	26.9 (16–41.2)	1	0.89–1.13	0.929
Base excess in blood (BE) (mmol/L)	1.8 (−13–16.6)	2.2 (−13–14.2)	1.2 (−8.7–16.6)	1.02	0.91–1.15	0.744

^1^ Significant.

## Data Availability

The original contributions presented in this study are included in the article material. Further inquiries can be directed to the corresponding author(s).

## Abbreviations

The following abbreviations are used in this manuscript:

PjP	*Pneumocystis jirovecii* pneumonia
HIV	Human Immunodeficiency Virus
EORTC/MSGERC	European Organization for Research and Treatment of Cancer/Invasive Fungal Infections Cooperative Group/Mycoses Study Group Education and Research Consortium
PCR	Polymerase chain reaction
